# Health professionals' perceptions about the decision-making process in the care of pediatric patients

**DOI:** 10.5935/0103-507X.20160057

**Published:** 2016

**Authors:** Eliana de Andrade Trotta, Fernanda Cristina Scarpa, Michel George El Halal, José Roberto Goldim, Paulo Roberto Antonacci Carvalho

**Affiliations:** 1 Faculdade de Medicina, Universidade Federal do Rio Grande do Sul - Porto Alegre (RS), Brazil. (in memorian); 2 Pediatric Intensive Care Unit, Hospital de Clínicas de Porto Alegre - Porto Alegre (RS), Brazil.; 3 Postgraduate Course in Child and Adolescent Health, Universidade Federal do Rio Grande do Sul - Porto Alegre (RS), Brazil.; 4 Department of Bioethics, Hospital de Clínicas de Porto Alegre - Porto Alegre (RS), Brazil.

**Keywords:** Patient care, Decision making, Intensive care units, pediatric, Ethics, professional, Resuscitation orders/psychology, Surveys and questionnaires, Coercion

## Abstract

**Objective:**

To evaluate the perceptions of physicians, nurses and nursing technicians of their participation in the decision-making process surrounding life support limitation in terminally ill pediatric patients, with comparisons by professional category.

**Methods:**

A cross-sectional study was conducted in the pediatric intensive care unit of a tertiary public university hospital with the participation of physicians, nurses and nursing technicians. The MacArthur Admission Experience Survey Voice Scale was used to assess and quantify the perceptions of professionals who assisted 17 pediatric patients with life support limitation within 24 hours after the outcome of each patient was determined. All professionals working in the unit (n = 117) who were potentially eligible for the study received a free and informed consent form prior to the occurrence of the cases studied.

**Results:**

Study participants included 25/40 (62.5%) physicians, 10/17 (58.8%) nurses and 41/60 (68.3%) nursing technicians, representing 65% of the eligible professionals identified. The questionnaire return rate was higher for physicians than technicians (p = 0.0258). A perceived lack of voice was reported in all three professional categories at varying rates that were lower for physicians than for nurses and nursing technicians (p < 0.00001); there was no difference between the latter (p = 0.7016). In the three professional categories studied, three subscale items were reported. For two of the three statements, there were significant differences between physicians and nurses (p = 0.004) and between physicians and nursing technicians (p = 0.001). For one of the statements, there was no difference among the three professional categories.

**Conclusion:**

Respondents perceived a lack of voice in the decision-making process at varying rates across the three categories of studied professionals who assisted terminally ill pediatric patients with life support limitation, with physicians expressing lowered rates of perceived coercion.

## Abstract (pt)

**Objetivo:**

Avaliar as percepções de médicos, enfermeiros e técnicos de enfermagem sobre sua participação no processo de tomada de decisão de limitação de suporte de vida, em pacientes pediátricos terminais, comparando por categoria profissional.

**Métodos:**

Estudo transversal realizado em unidade de terapia intensiva pediátrica de hospital público universitário, terciário, com a participação de médicos, enfermeiros e técnicos de enfermagem. Foi usada a Escala de Voz da *MacArthur Admission Experience Survey* para avaliar e quantificar a percepção dos profissionais que assistiram 17 pacientes pediátricos em limitação de suporte de vida, nas primeiras 24 horas após o desfecho de cada paciente. Todos os profissionais que atuavam na unidade (n=117), potencialmente elegíveis para a pesquisa, receberam o Termo de Consentimento Livre e Esclarecido previamente à ocorrência dos casos.

**Resultados:**

Participaram 25/40 (62,5%) médicos, 10/17 (58,8%) enfermeiros e 41/60 (68,3%) técnicos de enfermagem, representando 65% dos profissionais elegíveis. A taxa de devolução dos questionários pelos médicos foi maior que a dos técnicos (p = 0,0258). Houve registro de percepção de falta de voz nas três categorias profissionais, em taxas variáveis, porém menos percebida pelos médicos do que pelos enfermeiros e técnicos (p < 0,00001); entre estes últimos, não houve diferença (p = 0,7016). Nas três categorias profissionais, foram assinalados os três itens que compõem a subescala. Em duas das três afirmativas, houve diferença significativa entre médicos e enfermeiros (p = 0,004), e entre médicos e técnicos (p = 0,001). Em uma das afirmativas, não houve diferença entre as três categorias profissionais.

**Conclusão:**

Houve percepção de falta de voz no processo de tomada de decisão, em taxas variáveis, nas três categorias de profissionais que assistiram pacientes pediátricos terminais em limitação de suporte de vida, sendo os médicos os que expressaram menor percepção de coerção.

## INTRODUCTION

Since the 1970s, life support limitation (LSL) has been discussed as a way to provide a dignified death to patients with no therapeutic possibilities. After 20 years, LSL has become the most frequent way by which patients die in pediatric intensive care units (ICU) in the United States, Canada and several European countries.^(1-3)^

In 2008, after the International Consensus Conference on End-of-Life Care recommended that decision-making about end-of-life care should be shared between the physician and the patient or his/her family, the American College of Critical Care Medicine extended their recommendation that decisions center on the family and be shared with the multiprofessional team.^(4)^ Shared decision-making has been adopted by Canadian and American ICU, in which the participation of family members has been reported in more than 85% of cases.^(5,6)^ In the pediatric ICU of several countries in southern Europe and South America, a paternalistic model of decision-making is predominant, with decisions centered on the physician and low levels of participation by families and nurses.^(7-13)^

The exclusion of the nurse and differences of opinion between physicians and nurses concerning who should make decisions were observed in different countries^(14-17)^ and identified as causing discomfort for the professionals who do not participate in discussions but must perform the assistance as planned.^(18-20)^ In decision-making, a perceived lack of voice, in the sense of having the right to express an opinion, can be expressed and measured by instruments such as the scales of the MacArthur Admission Experience Survey,^(21,22)^ which have been formulated and validated for the assessment of Perception of Coercion, Voice and Procedural Justice in patients hospitalized in medical and surgical areas.^(23)^

The objective of this study was to evaluate and quantify the perception of voice by medical and nursing staff in a pediatric intensive care unit of a tertiary hospital during the process of life support limitation in terminally ill pediatric patients using a voice perception assessment questionnaire, relating the degree of perception of voice to the professional category of the individual surveyed.

## METHODS

This was a cross-sectional study that included physicians, nurses and nursing technicians in a pediatric medical-surgical ICU with 13 beds in a public tertiary university hospital in southern Brazil, encompassing the period from May 1, 2009, to May 30, 2010. All 117 professionals working in the unit who were potentially eligible for the study (40 physicians, including 14 intensive care physicians and 26 residents; 17 nurses; and 60 nursing technicians) received a free and informed consent form (FICF) prior to the occurrence of the cases studied. The project was approved by the Research Ethics Committee of the *Hospital de Clínicas de Porto Alegre*, under nº 08-210.

The index cases were 17 patients in whom there was LSL during the study period. The LSL included the addition of no new therapies, the decision to provide no resuscitation and the withdrawal of painful or unpleasant procedures considered useless. There was no withdrawal of previously established treatments. Sedation, analgesia, hydration and nutritional support were not excluded.

In each case, the physicians, nurses and nurse technicians who participated in the decision-making process and in the care of patients in LSL, from the point of initiation of LSL, or "zero time", to the determination of outcome, were identified. The "zero time" was considered to be the family meeting when LSL and end-of-life care decisions were determined. The expected outcomes were death, patient status change with withdrawal of LSL or discharge from the ICU to palliative care.

When a patient's outcome was determined, the Voice Scale questionnaire was made available to the identified professionals, preferably within 24 hours. The questionnaires were anonymous, with only the professional category of the respondent identified.

To minimize possible coercion or breach of confidentiality, the researchers asked all unit professionals to return their FICF in a sealed opaque collection box. Similarly, after the outcome of each patient in LSL was determined, the envelope with the survey instrument was made available to the designated participants in a predetermined location, wherein a sealed opaque collection box was placed for returning the envelope. At that stage, the collection box was opened only after the end of the survey period, at the time of data analysis.

The survey instrument (Voice Scale derived from the MacArthur Admission Experience Survey) consisted of three statements, which the respondents marked according to their agreement^(24)^ (Table 1).

**Table 1:** MacArthur Admission Experience Survey Voice Scale

Statements	Agree	Disagree
1. I had enough of a chance to say whether I agreed with the therapeutic limitation and ‘no resuscitation’ decision		
2. I got to say what I wanted about the therapeutic limitation and ‘no resuscitation’ decision		
3. My opinion about the therapeutic limitation and ‘no resuscitation’ decision did not matter		

Source: Taborda JG, Baptista JP, Gomes DA, Nogueira L, Chaves ML. Perception of coercion in psychiatric and nonpsychiatric (medical and surgical) inpatients. Int J Law Psychiatry. 2004;27(2):179-92.

The data were analyzed using Statistical Package for the Social Sciences (SPSS) version 18 and WINPEPI version 10.11.^(25)^ The chi-square test with Pearson's correlation and the multiple-comparisons test for proportions with Bonferroni correction were performed. The significance level adopted was 5% (p < 0.05).

## RESULTS

There were 633 hospitalizations during the study period, with 62 deaths (9.8%). Of these, 17 (27.4%) patients were considered to have exhausted therapeutic possibilities, constituting the index cases. In all cases, LSL, including a 'no resuscitation' decision, occurred.

The median age of the patients was 53 months (interquartile range [IQ]: 6 - 106). The median time between the decision and the outcome was 21 hours (IQ: 8.25 - 66.5). The team of caregivers eligible for the study consisted of 40 physicians (14 intensive care physicians and 26 rotating residents), 17 nurses and 60 nurse technicians. Women predominated in all three categories (100% of the nurses, 77.5% of the physicians and 98.3% of the technicians).

The overall return rate of the FICF was 65%, and 25/40 (62.5%) surveys returned corresponded to physicians, 10/17 (58.8%) to nurses and 41/60 (68.3%) to nursing technicians, with no significant difference among the three categories of caregivers. The 76 professionals who returned the FICF were considered the subjects of the study.

A total of 376 data collection instruments were distributed among the professionals involved with the patients in LSL and 227 (60%) of them were returned. Two instruments, one from a physician and one from a nursing technician, were excluded due to incomplete data. The return rate of the questionnaires was 65% by physicians, 61% by nurses and 52% by nursing technicians. There was no significant difference in the return rate of instruments between physicians and nurses, and between nurses and nursing technicians. However, there was a significant difference in the return rate between physicians and nursing technicians (p = 0.0258). These results do not interfere with the quality of the data obtained.

The lack of possibility to express an opinion, measured by the Voice Scale, showed a significant association (p < 0.00001) with the answers given by both nurses and nursing technicians, compared to physicians (Table 2). There was no difference in the lack of voice when comparing responses among nurses and nursing technicians (p = 0.7016).

**Table 2:** General analysis of the results obtained from the Voice Scale to evaluate the expression of a lack of possibility to express an opinion in the care decision-making process

Category	Respondents N	Lack of possibility to express opinion N (%)
Physicians (P)	120	17 (14)
Nurses (N)	50	38 (76)
Nursing technicians (NT)	55	40 (72)

Comparisons: P *versus* N: p < 0.00001; P *versus* NT: p < 0.00001; N *versus* NT: p > 0.05 (not significant).

The three statements in the questionnaires were analyzed individually for each professional category. A lack of possibility to express an opinion was mentioned on all items in the three professional categories, at variable rates.

The Voice Scale items indicated a significant association between nurses and nursing technicians with respect to physicians regarding the perception of not having had enough opportunity to say whether they agreed with the therapeutic limitation or with the 'no resuscitation' decision (item 1), as well as not having had the opportunity to express their wishes concerning that subject (item 2). There was no association among the professional categories regarding the perception that their opinions about therapeutic limitation or 'no resuscitation' decisions did not matter (Table 3).

**Table 3:** Evaluation of the responses to the items on the Voice Scale

Item	Physicians (P) N (%)	Nurses (N) N (%)	Nursing technicians (NT) N (%)	p value*
1. I had enough of a chance to say whether I agreed with the therapeutic limitation and no resuscitation	3 (17.6)	24 (63.2)	31 (79.4)	0.004 (P *versus* N) 0.001 (P *versus* NT)
2. I got to say what I wanted about the therapeutic limitation and no resuscitation	6 (35.3)	31 (81.5)	34 (87.1)	0.005 (P *versus* N) 0.002 (P *versus* NT)
3. My opinion about the therapeutic limitation and no resuscitation did not matter	14 (82.3)	35 (92.1)	32 (82)	NS

NS - not significant.

* Multiple-comparisons test for proportions with Bonferroni correction.

## DISCUSSION

In this study, the perceptions of physicians, nurses and nursing technicians who participated in the care of 17 children in LSL were analyzed. Instead of fictitious situations or questionnaires, which may not be representative of reality, we opted to use real cases involving patients in the intensive care unit. This decision was made to address the fact that feelings can differ according to the characteristics, illness and family of a patient.^(26)^

The analysis of responses to the three statements that constitute the Voice Scale indicated that the three classes of professionals studied perceived a lack of opportunity to express their opinions about decisions regarding their patients. In a study conducted by Lind et al., the expression of opinions and the validation of decisions were studied in hospitalized adult patients. Patients had the perception that they were not able to express their opinions or that their opinions were not seriously considered in the decision-making process.^(27)^ The data from the present study reiterate, from the perception of the professionals involved, the results obtained with patients.

The decision-making model in the studied pediatric ICU can still be considered predominantly paternalistic, despite signs of movement toward a more shared decision proposal.^(28)^ Decisions were not made exclusively by physicians, but the participation rates of nursing professionals were low during the period studied. Family members were always informed and involved in the decision-making process from the beginning of discussions regarding LSL. Participation mainly constituted accepting or rejecting the proposed options rather than making shared decisions. This result is similar to those reported in France.^(29)^ The same model has also been described in countries with cultures similar to Brazil such as Argentina,^(7)^ Portugal^(30)^ and Italy.^(31)^

The perceptions of nurses and physicians reported in national^(11,12)^ and international studies are very similar to the data obtained in this study. The same is not true regarding nursing technicians, given the lack of data from this particular professional category.^(32)^ However, the associations found between nurses and nursing technicians were always similar and differed from those obtained from doctors.

The limitations of this study were related to the fact that the perceptions reported represent professionals from a single pediatric ICU in southern Brazil. It is possible that the percentage of professionals who did not return the FICF (35%) represents a portion of the group as much or more uncomfortable with the situation.

The participation of other institutions as a control group could provide better insight into the perception of these professionals regarding the lack of voice in decision-making in the care of patients in LSL.

## CONCLUSION

This study demonstrated a perceived lack of opportunity to express an opinion during the care decision-making process using a validated instrument, the Voice Scale, to report on cases involving the end-of-life care of children. In the three professional categories studied, physicians expressed less of a perceived lack of opportunity to express their opinions compared to nurses and nursing technicians.
